# Witnessing Violence Toward Siblings: An Understudied but Potent Form of Early Adversity

**DOI:** 10.1371/journal.pone.0028852

**Published:** 2011-12-21

**Authors:** Martin H. Teicher, Gordana D. Vitaliano

**Affiliations:** 1 Department of Psychiatry, Harvard Medical School, Boston, Massachusetts, United States of America; 2 Developmental Biopsychiatry Research Program, McLean Hospital, Belmont Massachusetts, United States of America; Wayne State University, United States of America

## Abstract

Research on the consequences of witnessing domestic violence has focused on inter-adult violence and most specifically on violence toward mothers. The potential consequences of witnessing violence to siblings have been almost entirely overlooked. Based on clinical experience we sought to test the hypothesis that witnessing violence toward siblings would be as consequential as witnessing violence toward mothers. The community sample consisted of unmedicated, right-handed, young adults who had siblings (n = 1,412; 62.7% female; 21.8±2.1 years of age). History of witnessing threats or assaults to mothers, fathers and siblings, exposure to parental and sibling verbal abuse and physical abuse, sexual abuse and sociodemographic factors were assessed by self-report. Symptoms of depression, anxiety, somatization, anger-hostility, dissociation and ‘limbic irritability’ were assessed by rating scales. Data were analyzed by multiple regression, with techniques to gauge relative importance; logistic regression to assess adjusted odds ratios for clinically-significant ratings; and random forest regression using conditional trees. Subjects reported witnessing violence to siblings slightly more often than witnessing violence to mothers (22% vs 21%), which overlapped by 51–54%. Witnessing violence toward siblings was associated with significant effects on all ratings. Witnessing violence toward mother was not associated with significant effects on any scale in these models. Measures of the relative importance of witnessing violence to siblings were many fold greater than measures of importance for witnessing violence towards mothers or fathers. Mediation and structural equation models showed that effects of witnessing violence toward mothers or fathers were predominantly indirect and mediated by changes in maternal behavior. The effects of witnessing violence toward siblings were more direct. These findings suggest that greater attention be given to the effects of witnessing aggression toward siblings in studies of domestic violence, abuse and early adversity.

## Introduction

Studies on the clinical consequences of witnessing domestic or family violence have focused almost exclusively on the impact of observing violence between adults, and most specifically on witnessing violence toward mothers. This focus is reflected in and perpetuated by the instruments used to assess exposure. For example, the revised Conflict Tactics Scale, Parent-Child version [1], [2], which is the most frequently used instrument, has items to assess witnessing of violent acts between adults, but not between parents and siblings. So too does the ‘Things I've Seen and Heard’ survey [3]. The Adverse Childhood Experience Scale includes, as a key factor, witnessing the assault of mother or stepmother, but does not inquire about witnessing assaults of other family members [4], [5]. The detailed Child Exposure to Domestic Violence Scale (CEDV) [6], [7] has 17 items that inquire about the physical or emotional abuse of a child's mother by her partner, and 8 items that assess witnessing of violent or abusive events outside the home, but no items regarding witnessing of abuse towards siblings or father. Consequently, very little is known regarding the specific consequences of witnessing violence towards siblings.

One noteworthy exception is the Juvenile Victimization Questionnaire (JVQ) that was created by Finkelhor and colleagues [8], [9] to provide a more comprehensive assessment of exposure to violence in 8–17 year olds, and to rectify deficiencies in earlier instruments. The JVQ provides, possibly for the first time in a publicly available instrument, an item (in an optional module) about witnessing parental assault of siblings. Although the specific effects of exposure to this type of adversity were not emphasized, tabled results from bivariate analyses of 43 different types of adversity showed that witnessing parental assault of a sibling was associated with significant effects (p<0.01) on ratings of depression, anxiety and anger on the Trauma Symptoms Checklist for Children and Trauma Symptom Checklist for Young Children [9]. However, these effects were not corrected for exposure to other forms of adversity, nor directly contrasted with effects of witnessing violence toward other family members.

In the course of conducting research on the enduring effects of early adversity on brain development we included in our assessment instrument items about witnessing domestic violence as well as specific items about witnessing or hearing threats or assaults to mother, father and siblings. Our primary reason for including items about threats or assaults to siblings emerged from the senior author's experience treating a patient who reported witnessing the physical abuse of her sibling, and his impression that this was a pivotal event in the patient's life.

The aim of this study was to test the hypothesis that witnessing sibling assaults, or hearing siblings threatened during childhood, would be associated in early adulthood with effects on psychiatric symptom ratings that were as significant as witnessing violence towards mothers or fathers. Previous research has linked witnessing of interparental violence to a wide range of psychological, emotional, behavioral, social, and academic problems [10], [11], [12], [13], [14], [15], [16], [17], [18]. We found that witnessing violence towards siblings occurred as often as witnessing violence towards mothers in subjects with siblings, and there was about 50% overlap. To our surprise we found, after controlling for exposure to multiple forms of adversity, that witnessing violence toward siblings was associated with substantial effects on ratings of depression, anxiety, somatization, anger-hostility, dissociation and ‘limbic irritability’ while witnessing violence toward mother was not.

## Methods

### Ethics Statement

This Project has been reviewed and approved by the McLean IRB, Assurance # FWA00002744. During the review of this Project, the IRB specifically considered (i) the risks and anticipated benefits, if any, to subjects; (ii) the selection of subjects; (iii) the procedures for securing and documenting informed consent; (iv) the safety of subjects; and (v) the privacy of subjects and confidentiality of the data. All participants gave written informed consent prior to participation.

### Participants

Detailed ratings of symptoms and exposure to trauma, abuse, and witnessing violence towards mother, father and siblings were collected and analyzed from our multi-study community database which was collected between January 2004–January 2008. The database contains information from 1,662 healthy, unmedicated, right-handed, young adults (636 male and 1,026 female), 18–25 years of age, who responded to an advertisement entitled “*Memories of Childhood*.” Subjects were screened by phone for age, handedness, medications and general health. Subjects meeting these basic requirements were provided with a URL and password to a HIPAA-compliant online enrollment system, which collected detailed information on their life experiences, medical and psychiatric history, developmental history, demographics and psychiatric symptomatology (2,342 entry fields). Subjects were also given a contact number for a study psychiatrist who was available by page if a subject became distressed by the questions. None did. We focused on a group of 1,412 subjects (526 male and 886 female, 21.8±2.1 years of age) who had siblings. This sample was 75% White, non-Hispanic, 9% Black, non-Hispanic, 6% Asian, non-Hispanic, 4% other race, non-Hispanic, and 8% Hispanic, any race.

### Assessments

#### Abuse and trauma ratings

History of exposure to physical abuse was obtained by self-report to the question: *“Have you ever been physically hurt or attacked by someone such as a parent, another family member or friend (for example have you ever been struck, kicked, bitten, pushed or otherwise physically hurt)?”* If so, they were asked to provide information on their relationship to this individual, the number of times they were hurt, age of initiation and termination of these episodes, whether the abuse received, or should have received medical attention, and whether the abuse resulted in permanent injuries or scars [17]. An individual was defined as having experienced physical abuse if they reported any episode of inflicted physical injury that received or should have received medical treatment or resulted in permanent injury, or if they reported at least 4 episodes in which they felt that they had been attacked to a less serious degree.

Individuals were defined as having experienced sexual abuse if they responded affirmatively to the question: *“Have you ever been forced into doing more sexually than you wanted to do or were too young to understand? (By “sexually” we mean being forced against your will into contact with the sexual parts of your body or his/her body)”* [17]. They were also asked to provide information on their relationship to this individual, number of times they were forced, age of first and last abuse, and whether or not they felt terrified or had their life or another person's life threatened.

History of witnessing domestic violence was assessed using the questions: *“Have you ever witnessed serious domestic violence?” “Have you heard domestic violence in you family?” “Have you watched your mother (father, siblings) threatened or assaulted?”* and *“Have you heard your mother (father, siblings) threatened or assaulted?”* Ratings for seeing versus hearing threats or assaults overlapped from 94% (siblings) to 97% (fathers) and were combined into single items for seeing or hearing threats or assaults to mothers, fathers and siblings.

Ratings of exposure to parental or peer verbal abuse were assessed using the Verbal Abuse Questionnaire [17], which consists of 15 items that cover the key components of verbal abuse—scolding, yelling, swearing, blaming, insulting, threatening, demeaning, ridiculing, criticizing, screaming, belittling, and so on. In a preliminary sample of 48 college students, the Verbal Abuse Questionnaire showed high internal consistency as applied to reports of both maternal behaviors (Cronbach alpha = 0.98) and paternal behaviors (Cronbach alpha = 0.94). In the present sample, the Verbal Abuse Questionnaire also showed high internal consistency for sibling verbal abuse (Cronbach alpha = 0.96 and 0.97 for female and male siblings, respectively). Cut scores were used to dichotomize response [17], [19], [20] so that the impact of exposure to verbal abuse could be compared more directly to exposure to other forms of abuse that were rated as present or absent.

#### Symptom ratings

Self-report ratings were obtained using Kellner's Symptom Questionnaire [21], the Dissociative Experience Scale [22], and the Limbic System Checklist–33 [23]. The Kellner Symptom Questionnaire provides four symptom scales (depression, anxiety, anger-hostility, and somatic complaints). Depression and anxiety scores ≥12 are considered clinically significant [21]. Dissociative Experience Scale scores >30 are considered clinically significant and warrant further investigation [24]. The Limbic System Checklist–33 evaluates the frequency with which participants experience symptoms often encountered as ictal temporal lobe epilepsy phenomena [25]. Scores ≥40 are considered clinically significant [20].

#### Demographic characteristics

Data on race, ethnicity, education, parental education, family income, and perceived financial sufficiency during childhood (1 = much less than enough money to meet our needs, 5 = much more than enough money to meet our needs) were collected. We included perceived financial sufficiency as an alternative to family income, as participants were often uncertain of their parents' income during childhood, and family income could mean very different things depending on locale, family size, and parental spending habits. In all cases, perceived financial sufficiency explained a greater share of the variance in symptom ratings than family income.

### Data Analysis

#### Statistics

Exposure to one form of early adversity is frequently accompanied by exposure to other forms of adversity [5], [9], [26]. Hence, we used, in our primary statistical approach, general linear model regression techniques (ANCOVA / multiple linear regression) to estimate main effects of witnessing maternal, paternal and sibling violence while controlling for exposure to different forms of adversity and sociodemographic factors.

This approach assumes that there is an additive relationship between exposure to different types of adversity. This is a reasonable assumption as the Adverse Childhood Experience Study has shown an essentially additive ‘dose-related’ effect between exposure to multiple forms of early adversity and ratings of depression, suicide attempts, drug and alcohol use and receipt of psychotropic drugs [27]. Green et al [28] has also shown an essentially additive relationship in a nationally representative sample of adults. In addition to effects related to witnessing violence towards mother, father and siblings, the model included as covariates exposure to sexual abuse, parental and sibling physical abuse, parental and sibling verbal abuse, and socioeconomic factors in the form of parental education and perceived financial sufficiency during childhood. Regression fits were assessed for outliers (total n = 13 across the 6 scales) and for values with excess leverage (ca. 3 per scale), constituting about 0.2% of the data, which were excluded. Quantile-comparison plots of the studentized residuals were used to check for normality of distributed errors, which was met to a satisfactory degree, and spread level plots were used to check for heteroscedasticity, which was modest (<3 SD spread). Analyses of transformed data to further limit heteroscedasticity produced the same results in terms of significant regressors and relative effect sizes. Results from non-transformed analyses are presented as they are more readily understandable. The final regression models consisted of regression coefficients for witnessing maternal, paternal and sibling violence and covariates that had at least a marginal association (p<0.2) with the dependent variable.

Logistic regression analyses, with the same pallet of possible covariates, were conducted to ascertain the adjusted odds ratio, with 95% confidence index, for witnessing of violence toward mother, father and siblings on clinically-significant ratings of depression, anxiety, dissociation and limbic irritability.

#### Relative Importance

State-of-the-art techniques have been developed in recent years to more accurately gauge the relative importance of the individual regressors in a multiple regression. Johnson and Lebreton [29] define relative importance as *“the proportionate contribution each predictor makes to r^2^, considering both its direct effect (i.e., its correlation with the criterion) and its effect when combined with the other variables in the regression equation”.* Assessment of relative importance in linear models is simple in the special case where all regressors are uncorrelated. Each regressor's contribution then is their univariate r^2^, and all univariate r^2^-values add up to the full model r^2^. This is rarely true with observational data. Regressors are typically correlated, so that it is no longer straightforward to break down model r^2^ into shares from the individual regressors [30]. Hence, we used a technique for variance decomposition developed by Lindeman, Merenda, and Gold [31], and recommended by Johnson and Lebreton [29] and Grömping [30] to more accurately gauge the relative importance of exposure to witnessing violence toward mother, father or sibs. Briefly this technique decomposes r^2^ by calculating the sequential contribution of each regressor (in which the contribution of a regressor depends on the regressors that come before) by averaging over all possible sequential orderings (R package *relaimpo*). Similarly, logistic regression analyses were analyzed using a penalized lasso procedure to diminish or eliminate the contribution of correlated regressors (R package *glmnet* [32]).

#### Random Forest Regression

Random forest regression was used as a novel alternative statistical technique to assess the relative importance of exposure to witnessing violence toward mother, father or siblings on the measures of interest. Random forest regression is a modern analytical approach, primarily used for data mining that is not bound by the same assumptions as linear regression. Random forest regression was developed by Breiman [33] as an extension of the decision tree approach. It is a form of “ensemble learning” in which a very large number of small unpruned decision trees are generated and their results aggregated. This technique performs very well compared to many other classifiers, including discriminant analysis, support vector machines and neural networks [34], provided that predictor variables are similar in their scale of measurement or number of categories [35]. We used a variant of Breiman's approach which generates conditional trees to avoid a potential problem with biased estimates that emerges when variables differ in range or number of categories (‘*cforest*’ in R package *party* [35]). For these analyses 500 trees were generated with 3 variables randomly selected for evaluation at each node. Conditional forest regression indicates importance by assessing the increase in mean square error of the forest's fit following the permutation (effective elimination) of a given predictor variable. The more the permutation of a variable increases mean square error the greater the importance of the variable.

Random forest models included variables for witnessing maternal, paternal and sibling violence, sexual abuse, parental and sibling physical abuse, parental and sibling verbal abuse, parental education and perceived financial sufficiency.

#### Mediation

**Figure 1:**
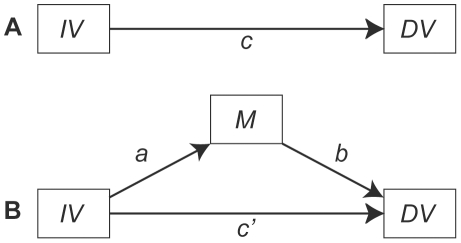
Diagram of classic mediation model. Panel A illustrates the total effect of the independent variable (IV) on the dependent variable (DV) as measured by regression coefficient *c*. Panel B shows the indirect effect of IV on DV via mediator M. The indirect effect is quantified as path *a* (IV→M) times path *b* (M→DV) *or ab*. The director effect is indicated by c′.

Mediation analyses were used to ascertain the degree to which potential effects of exposure to witnessing violence toward mother, father or siblings were mediated through indirect effects stemming from increased levels of maternal or sibling verbal aggression toward the subject. Figure 1 shows the classic single variable mediation model in which the total effect of the independent variable on the dependent variable (path *c*) is mediated indirectly through variable M via paths *a* and *b*, and directly through path *c*′. Traditionally, mediation is detected through the causal steps approach popularized by Baron and Kenny [36], and/or by the Sobel test [37] to evaluate the significance of path coefficient *a* multiplied by path coefficient *b (ab)* [38]. The causal step approach has recently been criticized because simulation studies have shown that this approach is amongst the least powerful method for testing intervening variable effects [39], [40]. The Sobel test also has a significant flaw. It requires the sampling distribution of the indirect effect *ab* to be normal, though it tends to be asymmetric, with nonzero skew and kurtosis [38], [41]. Simulation research shows that modern bootstrap-based methods are more powerful than the Sobel test and the causal steps approach [42], [43]. Bootstrapping methods were implemented in R (‘*mediation*’ in R package *MBESS* [44]) to calculate *a*, *b*, *c* and *c′*, with p values, the indirect effect (*ab*) with 95% confidence intervals, ratio of indirect to total effect [45] also known as mediation ratio [44], ratio of indirect to direct effect [37] and the shared over simple effects (SOS) index, which is the ratio of the variance in Y explained by both X and M divided by the variance in Y explained by X [46].

Path analysis (R package *OpenMx*) was used to evaluate structural equation models showing the interrelationship between exposure to various forms of maltreatment (witnessing violence towards mother, father or siblings, sexual abuse), psychiatric symptom ratings and potential mediators. Goodness of fit was evaluated using a combination of absolute fit and relative fit indices to minimize Type I and Type II errors [47]. Absolute fit was evaluated by χ^2^ and standardized root mean square residual (SRMR). A significant χ^2^ indicates that the model can be rejected. However, χ^2^ is strongly influenced by sample size and structural equation models with large n's (>200) are usually significant and may be rejected unfairly. SRMR is not as strongly influenced by sample size and values less than 0.08 are indicative of a good fit [47]. Relative fit indices include the Normed Fit Index (NFI), Tucker-Lewis Index (TLI), Comparative Fit Index (CFI) and Incremental Fit Index (IFI), with the later being the least sensitive to sample size [48]. Relative fit indices with values greater than 0.95 are indicative of good fits.

## Results

**Figure 2:**
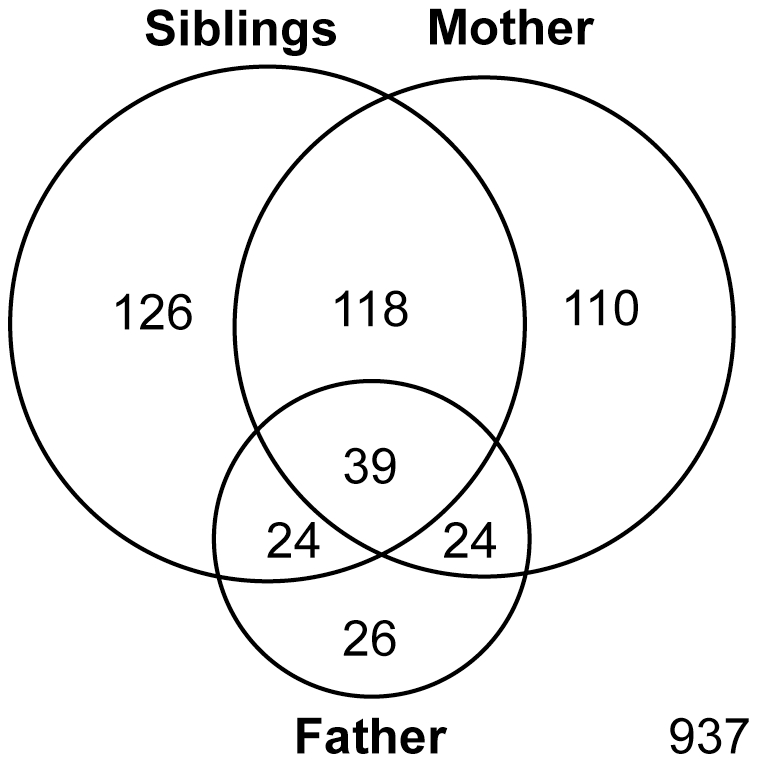
Venn diagram. Overlapping circles indicating the number of subjects who witnessed threats or assaults to mother, father or sibling and the degree of co-occurrence. There were 937 subjects in the sample who witnessed no threats or assaults to family members.

Altogether, 291 (21%), 113 (8%) and 308 (22%) subjects, from the sample of 1412, reported seeing or hearing threats or assaults to their mother, father and siblings, respectively, at any time during their childhood. However, there was substantial overlap in types of exposure as seen in the Venn diagram (Figure 2). Fifty-four percent of subjects who reported witnessing violence toward mother reported witnessing violence toward siblings, and 22% reported witnessing violence toward father. Similarly, 51% and 21% of subjects reporting witnessing violence toward siblings reported witnessing violence toward mother and father, respectively. Fifty-six percent of subjects who witnessed violence toward father also witnessed violence toward siblings, and the same percent witnessed violence towards mothers.

Results of the multiple linear regression models were consistent (Table 1). Witnessing threats or assaults to siblings was associated to a significant degree with ratings of depression, anxiety, somatization, anger-hostility, dissociation, and ‘limbic irritability’. The effect size for witnessing violence towards siblings, as indicated by regression coefficients and confidence intervals, was comparable in magnitude to experiencing sexual abuse. Witnessing threats or assaults to father was not significantly associated with any of the symptom ratings except for somatization (p<0.01). Similarly, witnessing threats or assaults towards mother was not associated to a significant degree with symptom rating in any of these models.

**Table 1:** Regression coefficients and 95% confidence intervals for the association between exposure to multiple forms of maltreatment and socioeconomic factors on psychiatric symptom ratings.

	Symptom Ratings					
				Anger-	Limbic	
Regressors	Depression	Anxiety	Somatization	Hostility	Irritability	Dissociation
Witness Violence- Mother	−0.216	0.035	0.003	0.060	−0.063	−0.959
	[−0.97–0.53]	[−0.66–0.73]	[−0.69–0.70]	[−0.66–0.78]	[−2.13–2.01]	[−2.26–0.34]
Witness Violence- Father	0.769	0.721	1.485**	0.882	1.852	1.545
	[−0.23–1.76]	[−0.20–1.64]	[0.55–2.42]	[−0.09–1.86]	[−0.89–4.59]	[−0.15–3.24]
Witness Violence- Sibs	1.437§	0.919**	1.274¥	1.235¥	5.407§	2.399¥
	[0.72–2.16]	[0.25–1.59]	[0.61–1.94]	[0.54–1.93]	[3.39–7.43]	[1.15–3.65]
Parental Verbal Abuse	2.224§	2.022§	1.675§	1.360§	5.062§	4.101§
	[1.58–2.87]	[1.42–2.62]	[1.07–2.28]	[0.69–2.03]	[3.24–6.89]	[2.97–5.23]
Sibling Verbal Abuse	–	–	0.430	0.776*	4.618§	1.811**
			[−0.18–1.04]	[0.14–1.41]	[2.75–6.49]	[0.65–2.98]
Parental Physical Abuse	–	–	–	0.814*	–	–
				[0.05–1.58]		
Sibling Physical Abuse	–	–	–	–	2.688**	1.342*
					[0.75–4.63]	[0.14–2.54]
Sexual Abuse	1.501§	1.353§	1.375§	–	5.440§	1.873**
	[0.85–2.15]	[0.74–1.96]	[0.77–1.98]		[3.61–7.27]	[0.74–3.00]
Parental Education	–	–	−0.092	−0.187¥	–	−0.196*
			[−0.19–0.00]	[−0.28–−0.09]		[−0.37–−0.02]
Financial Sufficiency	−0.830§	−0.444**	−0.303	–	−1.219*	−0.812*
	[−1.18–−0.48]	[−0.77–−0.12]	[−0.63–0.03]		[−2.18–−0.26]	[−1.43–−0.19]
Gender	–	0.584*	0.711**	–	−1.930*	−1.579¥
		[0.08–1.09]	[0.21–1.21]		[−3.44–−0.42]	[−2.51–−0.64]
Multiple R2	0.132†	0.112†	0.140†	0.098†	0.182†	0.154†

*p<0.05,

**p<0.01,

¥ p<0.001,

§ p<0.0001,

† p<10^−15^.

— Covariates were excluded from the final model if their degree of association with the dependent variable was weak (p>0.2).

**Figure 3:**
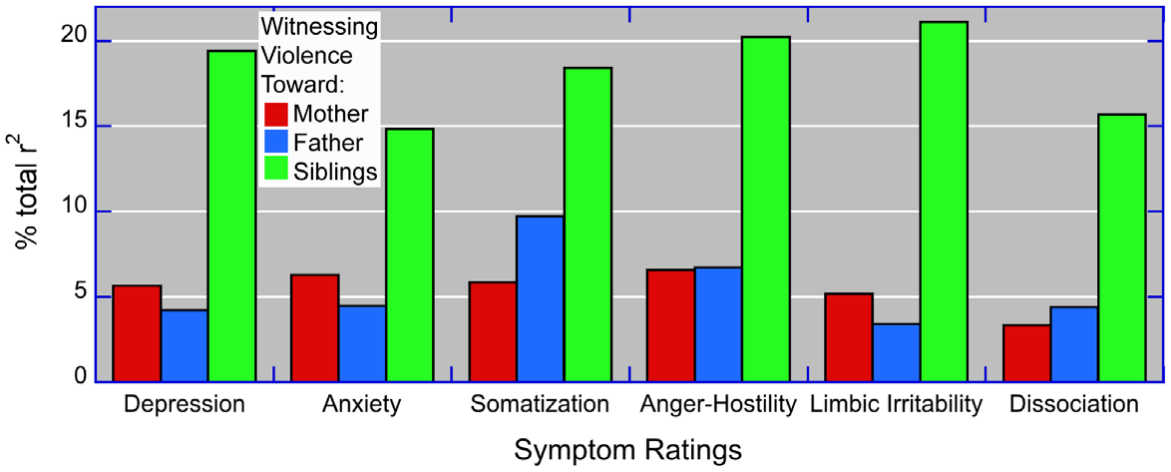
Relative importance – variance decomposition. Comparison of the relative importance of witnessing threats or assaults to mother, father or siblings based on regression analyses and variance decomposition with covariates (not shown) for sexual abuse, parental and sibling verbal abuse, parental and sibling physical abuse, sex and demographic factors.

Assessment of the relative importance of exposure to each form of domestic violence on symptom scores, using the technique of Lindeman et al [31], is shown in Figure 3.

According to these analyses witnessing threats or assaults to siblings accounted for 2.4–4.7-fold greater share of the total variance than witnessing threats or assaults to mother. Witnessing threats or assaults to father was similar in importance to witnessing threats or assaults to mother on most variables except for ratings of somatization, where it accounted for a 66% greater share of the variance.

Logistic regression analysis painted a similar picture (Table 2). Witnessing violence toward siblings was associated with significant adjusted odds ratios, which ranged from a low of 1.45 [95% CI 1.01–2.09] for anxiety to a high of 2.28 [95%CI 1.48–3.50] for clinically significant ratings of ‘limbic irritability’. Adjusted odds ratios for witnessing violence to siblings were comparable to adjusted odds ratios for experiencing sexual abuse. Witnessing violence toward mothers or fathers were not associated with statistically significant adjusted odds ratios. More detailed analysis using a penalized lasso technique designed to identify the most significant contributing variables eliminated regression coefficients for witnessing violence to mother or father but provided similar adjusted odds ratios for witnessing violence to siblings (data not shown).

**Table 2:** Adjusted odds ratios and 95% confidence intervals for the association between maltreatment, sociodemographic factors and clinically-significant psychiatric symptom ratings.

Regressors	Depression	Anxiety	Dissociation	Limbic Irritability
Witness Violence to Mother	1.07 [0.73–1.57]	0.92 [0.63–1.35]	1.14 [0.61–2.11]	1.14 [0.73–1.78]
Witness Violence to Father	1.30 [0.8–2.1]	1.51 [0.94–2.41]	1.52 [0.75–3.08]	1.16 [0.67–2.03]
Witness Violence to Siblings	1.69 [1.17–2.44]**	1.45 [1.01–2.09]*	1.86 [1.01–3.42]*	2.28 [1.48–3.5]¥
Parental Verbal Abuse	1.92 (1.35,2.74)¥	2.56 [1.86–3.53]¥	2.74 [1.55–4.85]¥	2.28 [1.52–3.42]¥
Sibling Verbal Abuse	–	–	–	1.73 [1.14–2.6]**
Parental Physical Abuse	1.36 [0.92–2.02]	–	–	–
Sibling Physical Abuse	–	–	–	1.76 [1.16–2.67]**
Sexual Abuse	1.85 [1.33–2.57]¥	1.59 [1.14–2.22]**		1.95 [1.3–2.94]¥
Parental Education	–	–	0.92 [0.83–1.01]	–
Financial Sufficiency	0.77 [0.63–0.93]**	0.81 [0.67–0.97]*	0.66 [0.47–0.93]*	–
Gender	–	1.33 [0.97–1.83]	0.63 [0.36–1.07]	0.77 [0.51–1.15]

*p<0.05,

**p<0.01,

¥ p<0.001.

– Covariates were excluded from the final model if their degree of association with the dependent variable was weak (p>0.2).

**Figure 4:**
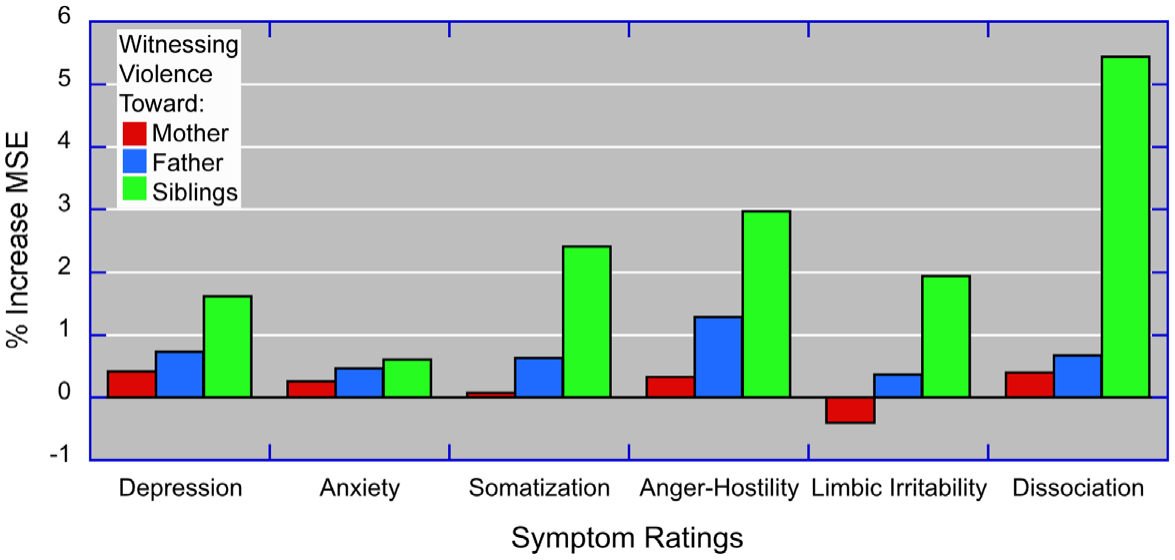
Relative importance – random forest regression. Comparison of the relative importance of witnessing threats or assaults to mother, father or siblings based on random forest regression using conditional trees with additional regressors (not shown) for sexual abuse, parental and sibling verbal abuse, parental and sibling physical abuse, sex and demographic factors.

Random forest regression (Fig. 4) revealed a consistent rank ordering of importance with witnessing threats or assaults to siblings>father>mother. Interestingly, witnessing threats or assaults to siblings was associated with high relative importance on symptoms of dissociation. None of these three types of adversity appeared to have a substantial relative impact on ratings of anxiety. In contrast, exposure to parental verbal abuse was associated with about a 10-fold greater impact on ratings of anxiety than witnessing violence toward siblings (results not shown).

The apparent low relative importance of witnessing threats or assaults to mother likely occurred because one or more of the other regressors in the model mediated the effects. It is also likely that witnessing violence towards siblings had high relative importance because the effects were more direct and mediated to a lesser degree by other regressors in the model. In particular, we predicted that the effect of witnessing violence toward mother would be mediated to a considerable degree by increased levels of maternal verbal aggression towards the subject. To test this hypothesis we assessed the total effect of witnessing threats or assaults to mother in a subsample that excluded subjects who witnessed violence toward fathers or siblings (n = 1047, 406M/641F). Similarly, we assessed the direct and indirect effects of witnessing threats or assaults to sibling in a subsample that excluded subjects who witnessed violence towards mothers or fathers (n = 1063, 403M/660F).

As shown in Table 3, there were significant total effects (*c*) of witnessing threats or assaults to mother on all symptom scores. There were also very strong relations between witnessing violence towards mother and receipt of maternal verbal abuse, and between maternal verbal abuse and symptom ratings. The direct effect (*c*′) was substantially smaller than the total effect and no longer reached conventional levels of significance. Standardized indirect effects ranged from 0.046 to 0.064, and were significant as their 95% confidence intervals did not include 0. The indirect effect mediated by maternal verbal abuse constituted about 50% of the total effect, and SOS Indices ranged from 0.731 to 0.815.

**Table 3:** Beta weights and comparative ratios indicating the role of maternal verbal aggression in mediating the association between symptom ratings and witnessing threats or assaults to mothers.

	Symptom Ratings					
Mediation				Anger-		Limbic
Measures	Depression	Anxiety	Somatization	Hostility	Dissociation	Irritability
Total Effect *c*	0.100¥	0.103¥	0.111§	0.103¥	0.087**	0.104¥
Direct Effect *c′*	0.041	0.046	0.048	0.049	0.043	0.047
IV→M *a*	0.228†	0.228†	0.228†	0.228†	0.228†	0.228†
M→DV *b*	0.260†	0.252†	0.278†	0.241†	0.193†	0.254†
Indirect Effect *ab*	0.058**	0.057**	0.064**	0.053**	0.046**	0.059**
Indirect Effect 95%CI	[0.04–0.08]	[0.04–0.08]	[0.04–0.09]	[0.03–0.08]	[0.03–0.07]	[0.04–0.09]
Indirect/Total	0.550	0.498	0.554	0.468	0.549	0.558
Indirect/Direct	1.223	0.991	1.241	0.881	1.218	1.261
SOS	0.808	0.761	0.811	0.731	0.808	0.815

*p<0.05,

**p<0.01,

¥ p<0.001,

§ p<0.0001,

† p<10^−10^.

DV – Dependent Variable, IV – Independent Variable, M – Mediator, SOS – Shared over simple effects index.

The effects of witnessing violence to father were not mediated by increased levels of paternal verbal aggression. Similarly, effects of witnessing threats or assaults to siblings were mediated to only a minor degree by increased levels of sibling verbal aggression (12–20% of the total effect). On the other hand, witnessing threats or assaults to father and siblings were strongly associated with levels of maternal verbal abuse.

**Figure 5:**
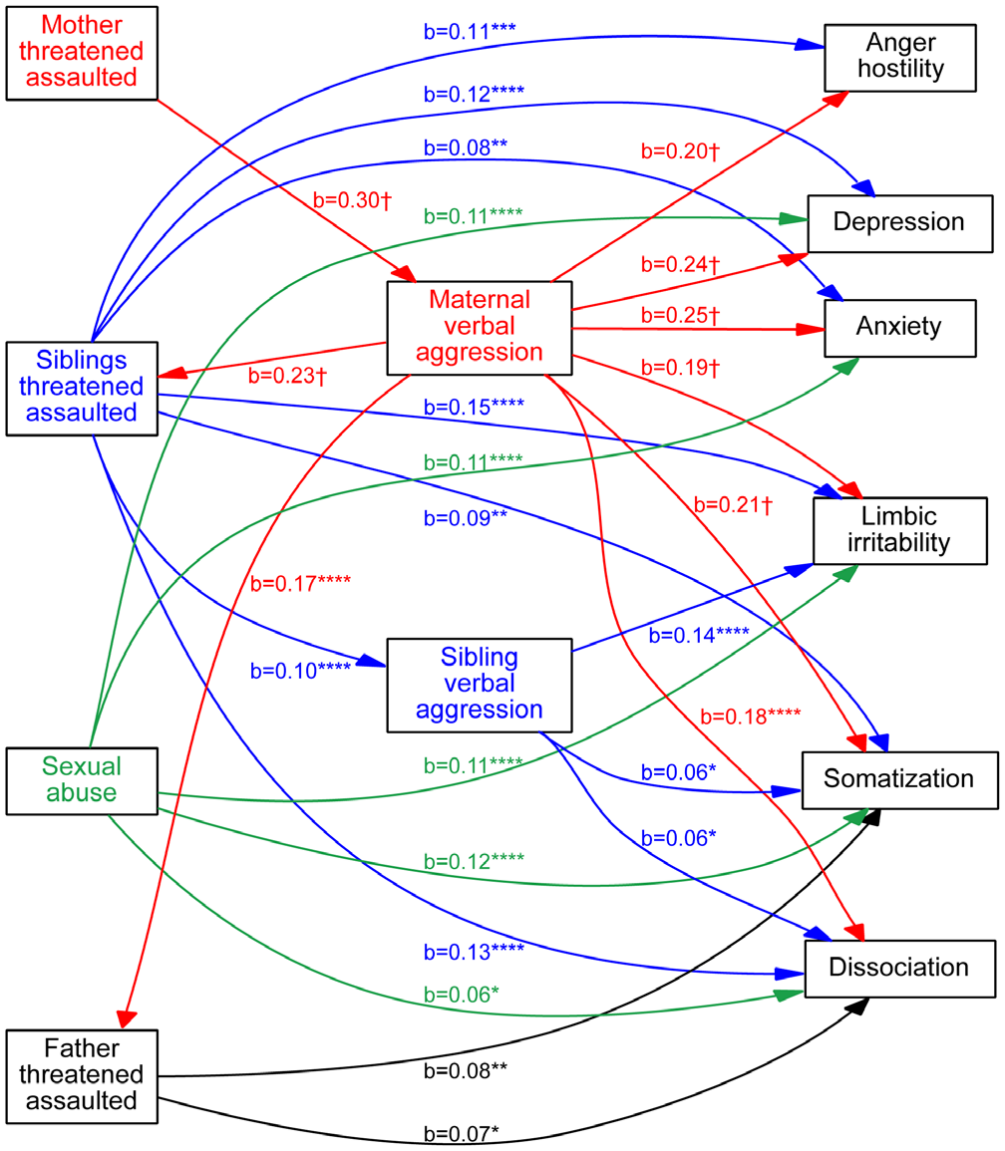
Path analysis. Best-fitting structural equation model showing the interrelationships between independent variables (witnessing violence toward mothers, fathers or siblings, childhood sexual abuse), dependent variables (ratings of depression, anxiety, somatization, anger-hostility, dissociation, limbic irritability) and mediators (maternal or sibling verbal aggression). Only significant paths are shown. Significant covariate relationships between variables of the same type were omitted for clarity. Paths are color-coded to indicate whether the origin of the arrow is from a variable associated with mother (red), father (blue), sibling (blue), or childhood sexual abuse (green). Standardized beta weights are provided with asterisks indicating significance levels. *p<0.05, **p<0.01, ***p<0.001, ****p<0.0001, †p<10^−10^.

The complex interrelationship between witnessing threats or assaults to family members and experiencing verbal abuse from parents or siblings on symptom ratings was modeled using path analysis. The best fitting model is diagrammed in Figure 5. The relationships proposed in the model provide a plausible explanation of those that exist in the data and could not be rejected by chi-square criteria (χ^2^ = 2.74, df = 4, p>0.6). The RMSR was 0.008 indicating a very good fit. Relative fit indices also indicated a very good fit (NFI = 0.999; TLI, CFI and IFI = 1).

The model included maternal and sibling verbal aggression as potential mediators. Attempts to include paternal verbal aggression in the model invariably resulted in much poorer fits. For clarity the model only shows significant paths between variables. Non-significant paths and covariance measures between variables of the same type have been omitted for clarity. There were significant paths from witnessing threats or assaults to siblings to ratings for all dependent variables. There was also evidence for a modest degree of mediation by sibling verbal abuse on ratings of ‘limbic irritability”, dissociation and somatization. Witnessing threats or assaults to father was associated with significant paths to somatization and dissociation. Sexual abuse (any perpetrator) was associated with elevated ratings for all dependent variables except anger-hostiity.

There were no significant direct paths from witnessing threats or assaults to mothers to symptom ratings. However, witnessing violence to mother had a strong influence on ratings of maternal verbal aggression, which was associated with marked effects on symptom ratings. Hence, the effect of witnessing violence toward mothers was mediated to a large degree by higher levels of maternal verbal aggression. There were also highly significant paths from maternal verbal abuse to witnessing of violence toward fathers and siblings, suggesting that in some instances that highly aggressive mothers may act violently toward other family members. Further, the apparent consequences of witnessing violence toward these family members (particularly fathers) on these subjects, could be an indirect consequence of exposure to maternal verbal abuse.

## Discussion

To the best of our knowledge this is the first study to specifically compare psychiatric symptoms associated with witnessing violence towards mother, father or siblings. The few previous studies that provided data on witnessing violence toward siblings reported that it occurred frequently, with about the same prevalence as witnessing adult partner violence. A Finish study reported that 12% of the adolescents (n = 1393) had witnessed interparental violence during their childhood, while 8% had witnessed father-to-sibling violence and 8% had witnessed mother-to-sibling violence [49]. A study of 15–17-year-old adolescents in Hong Kong (n = 415) found that 7.5% had witnessed adult partner violence while 9.2% had witnessed parental assault of a sibling [50]. A cross-sectional survey of 1,185 Palestinian secondary school students reported that 18.8% had witnessed their parents threaten their siblings with a knife, gun, stick, chair, or other injurious or lethal weapon while 18.4% had witnessed fathers do the same to mothers, and 7.7% witnessed mothers' respond in kind to fathers [51]. Moreover, 34.5% reported that they witnessed their parents attack their siblings continuously with a stick, club, or other harmful object at least once during their childhood, while 21.4% and 3.8% witnessed similar attacks of mothers by fathers and fathers by mothers, respectively [51]. The lower the quality of family housing, the more likely the participants were to report witnessing parent-to-sibling psychological and physical violence [51].

We found in the present study that witnessing violence toward siblings occurred 86% as often as witnessing violence toward mother in the entire sample (n = 1662), and 6% more often in the 1412 subjects who had siblings. Data from the National Survey of Children's Exposure to Violence (a representative telephone survey of 4,549 youth aged 0–17) showed that witnessing interparental violence was associated with a 5.55-fold increased in risk for witnessing sibling physical abuse during the last year, and with a 6.99-fold increase in risk of witnessing sibling physical abuse during their lifetime [52].

Very few studies have provided information on the potential consequences of witnessing violence toward siblings, and none controlled for exposure to other forms of adversity. Lepisto et al., (2011) reported that witnessing all forms of domestic violence were associated with self-perceived ill health and poor satisfaction with life [49]. Witnessing parent-to-parent violence and mother-to-sibling violence were risk factors for being bullied at school. The adolescent's role as a bully was correlated with witnessing domestic violence between mother and siblings [49]. Finkelhor et al. [9], found that witnessing parent assault of a sibling was associated with significant effects on ratings of depression, anxiety and anger in both younger and older children.

We found using multiple regression analyses, random forest regression and structural equation modeling, that witnessing violence towards siblings was associated with much greater effects on psychiatric symptom ratings than witnessing violence towards mother or father. This suggests that more attention should be given to the consequences of witnessing violence towards sibling both in research and in clinical practice. Indeed, the reason we chose to explore the impact of witnessing sibling abuse was due to its critical importance in the psychotherapy of a former patient.

The minimal apparent impact of witnessing violence toward mothers on psychiatric symptomatology in the present study is reasonably consistent with the literature. Kitzmann et al. [18], reported in a meta-analysis that the average effect size (Cohen's d) for exposure was 0.29, indicating a small effect, which varied inversely with the number of other forms of adversity controlled for. We controlled for more forms of adversity in the current study than is typical, and included two novel forms - exposure to parental verbal abuse and witnessing of violence towards siblings. Witnessing violence toward siblings is a significant confound that occurred along with witnessing violence toward mother in about 50% of incidents. Maternal verbal abuse in contrast was a major mediator.

These findings suggests that domestic violence toward mother affects the emotional well-being of her children by primarily altering her behavior, which may be reflected in her more frequent use of verbal aggression. Similarly, Henning et al [10] found that a substantial proportion of the variance accounted for in adult adjustment by interparental physical conflict was mediated through decreased parental caring and warmth during childhood.

In contrast the effects of witnessing threats or assaults to siblings were mediated to only a limited degree by changes in the siblings behavior towards the subject as indexed by the sibling's use of verbal or physical aggression. Rather the effects appeared to be more direct. Our supposition is that individuals who witnessed violence towards siblings, but were largely spared, suffered from ‘survivor's guilt’. Their guilt may be compounded if they tended to side with the abusive parent and shared in their sibling's maltreatment. It may also be the case that witnessing violence to siblings, but not necessarily experiencing the same, creates a persistent state of fear and uncertainty that may be more stressful then the actual event. Indeed, physical abuse by parents was not associated with significantly elevated symptom scores in the multiple regression and logistic regression analyses. We have found in pervious samples that exposure to physical abuse had weaker effects on these ratings than exposure to emotional maltreatment [17], [53], but greater effects on degree of drug and alcohol use [53].

Witnessing violence towards fathers was associates with significant effects on somatization scores in the regression models, and to somatization and dissociation ratings in the path analysis. We suspected that the effects of witnessing violence towards father on other rating scales were largely indirect, and that violence toward fathers was one manifestation of high levels of maternal aggression.

The study is limited as it is a cross-sectional analysis of a convenience sample, and it relies on retrospective self-report. Some critics have raised concern about recall bias, suggesting that subjects who are currently in emotional distress will describe their childhood as more stressful or abusive [54]. Others have raised concerns about false or ‘recovered’ memories [55] that arise during the course of psychotherapy or hypnotherapy. Based on these criticisms one might expect a high false positive rate for adult reports of childhood abuse. The opposite is actually the case. Evidence shows that there is a strong tendency for adults to under-report exposure. For instance, Williams [56] found that 38% of women with documented histories of sexual victimization (confirmed by ER visits at the time), did not recall the abuse when interviewed 17 years later, though they often recalled other instances. More recently Shaffer et al [57] confirmed in a group of subjects assessed both prospectively and retrospectively that subjects often minimize their degree of exposure on retrospective report. Consequently, there were significant problems with false negative but not false positive reports. Individuals reporting abuse retrospectively were those who typically endured the most severe abuse on prospective assessment. This fits with other studies showing that adult reports of abuse are verifiable [58]. Retrospective assessment was as at least as powerful as prospective assessment in delineating the psychiatric consequences of abuse [57]. This is reassuring, as thousands of papers have been published using retrospective reports of maltreatment on psychiatric symptoms, endocrinology and neurobiology.

The study is also limited, as we did not collect information on the family member(s) who threatened or assaulted the sibling, and did not collect separate information on threats versus assaults, or information on the frequency, severity and chronicity of the exposure. Replication in a Nationally-representative sample is needed, as are longitudinal studies and extensions with more definitive measures of exposure. This study however represents a reasonable first step that may motivate further research.

We need to emphasize that path analysis is a statistical tool that can apportion variance (path coefficients). We do not presume that it provides evidences for a causal relationship based on correlational data [59]. There are other potential alternative models and explanations though none seem as likely. One possibility is a passive genetic influence rather than an experiential effect. It is plausible to envision a sequence of polymorphisms that leads to an increased risk for abusive behavior by parents that could be inherited in part and associated with increased symptom ratings in the child. However, it is implausible that this genetic relationship would hold strongly when the target of the abuse was a sibling but not a parent, especially given that they often co-occur. Further, it is implausible that this series of risk genes would be present in ∼20% of US families. A second possibility is that children living in homes in which a sibling was abused were probably exposed to a host of other risk factors not directly related to violence. However, it is also likely that children living in homes in which a parent was abused were exposed to a similar or indistinguishable set of risk factors.

Overall, we believe that this study provides novel insight into the complex phenomenon of exposure to interfamilial violence. Our findings bring to light the possibility that witnessing violence towards siblings has a direct effect on symptom ratings, and may be a risk factor for mood, anxiety and dissociative disorders. Further, our findings suggest that the predominant focus of the field on violence toward mothers, or on adult partner violence, provides an incomplete perspective.
